# Theoretical and Experimental Investigations of High-Entropy (TiVNbTa)_2_AlC MAX Phase

**DOI:** 10.3390/ma19122593

**Published:** 2026-06-16

**Authors:** Lexing Che, Mingdong Bao, Zhihua Sun, Yingwen Cao

**Affiliations:** 1 School of Materials and Chemical Engineering, Ningbo University of Technology, Ningbo 315211, China; 2 School of Materials Science and Engineering, Chang’an University, Xi’an 710061, China

**Keywords:** MAX phases ceramics, high-entropy, first-principles calculation, electronic structure, tribological behavior

## Abstract

High-entropy MAX phases (TiVNbTa)_2_AlC have attracted increasing attention due to their potential advantages in structural stability, damage tolerance, and mechanical reliability under complex service environments. This work studied the crystal and electrical structures with the elastic properties, the synthesis reactions and further wear resistance of HE-MAX (TiVNbTa)_2_AlC theoretically and experimentally. The charge transfer between both M-C atoms and M-Al atoms turned more intense, which correspondingly strengthened the M-C and M-Al bonds, respectively. Because of the dope on M-sites, (TiVNbTa)_2_AlC exhibited larger fracture toughness *K_IC_* and a lower brittle index M, which suggested lower brittleness, better crack extension resistance, and higher damage tolerance than Ti_2_AlC. In this work, high-entropy (TiVNbTa)_2_AlC MAX phase ceramics were successfully synthesized by a powder metallurgy route combined with pressureless sintering and spark plasma sintering (SPS). The effects of raw material composition and sintering temperature on phase evolution, microstructure formation, mechanical properties, and tribological behavior were systematically investigated. The results show that a highly pure (TiVNbTa)_2_AlC phase with a phase fraction of 96.8% could be obtained at a molar ratio of M:Al:C = 2:1.2:0.8 and a sintering temperature of 1550 °C. Phase evolution analysis indicated that the reaction process followed the sequence of intermetallic compound (IMC) formation → carbide formation → MAX phase formation. Severe lattice distortion induced by the multi-principal-element solid solution significantly enhanced the hardness of the material, which was markedly higher than that of conventional ternary MAX phases. Owing to its higher hardness and more homogeneous solid-solution structure, HE-MAX (TiVNbTa)_2_AlC could inhibit the formation of surface microcracks and reduce the driving force for crack propagation to some extent. Therefore, the lower wear rate not only reflected improved tribological performance but also demonstrated the beneficial role of high-entropy design in enhancing resistance to surface damage.

## 1. Introduction

MAX phases are a class of ternary layered ceramics with the general formula M_n+1_AX_n_ (space group P6_3_/mmc) [1,2], in which M represents an early transition metal, A is an A-group element, and X is carbon and/or nitrogen [3,4]. The coexistence of strong M–X covalent bonds and relatively weak M–A metallic bonds results in a unique laminated structure consisting of alternating MX and A atomic layers along the c-axis [5]. Owing to this structural feature, MAX phases exhibit a unique combination of ceramic-like and metallic properties, including high strength, excellent thermal stability, good electrical and thermal conductivity, and machinability, making them promising candidates for high-temperature structural components, wear-resistant systems, and nuclear applications [6,7]. Recently, increasing demands for structural reliability in aerospace systems, high-speed friction systems, and complex loading environments have stimulated extensive interest in the damage evolution behavior of MAX phases under cyclic contact, thermo-mechanical coupling [8], and impact wear conditions [9,10].

However, conventional ternary MAX phases still exhibit several limitations. For example, although Ti_2_AlC possesses good thermal stability and damage tolerance, its relatively limited hardness and resistance to plastic deformation make it susceptible to interlayer slipping, surface spallation, and wear damage under cyclic contact loading, thermal friction, and local stress concentration conditions [11,12,13,14]. Existing research indicates a significant coupling relationship between microcrack initiation on friction surfaces and localized plastic deformation, with the material’s intrinsic hardness, lattice stability, and interfacial structure playing decisive roles in crack initiation and propagation behavior [15,16]. Therefore, improving the damage resistance and service reliability of MAX phase materials under complex stress states has become an important research direction in this field.

To overcome the performance bottlenecks of traditional MAX phases, the high-entropy design strategy has been widely applied in recent years [17,18,19]. This strategy introduces multiple equi-atomic transition metal elements at the M-site, utilizing severe lattice distortion effects, sluggish diffusion effects, and local chemical fluctuations to achieve synergistic strengthening, thereby significantly improving the material’s mechanical properties and high-temperature stability [20,21]. Research has shown that high-entropy design can markedly enhance the hardness, strength, and high-temperature stability of MAX phase materials [22,23]. For example, Wu et al. successfully developed the high-entropy MAX phase (TiZr_0.6_NbTa)_2_AlC, introducing local chemical fluctuations (LCF) into the atomic stacking layers of the MAX phase, ultimately enhancing plasticity at both room and elevated temperatures [24]. Chen et al., using thermogravimetric analysis, found that (V_0.2_Nb_0.2_Ta_0.2_Mo_0.2_W_0.2_)_2_AlC showed no volume change in air from 0 to 400 °C, and its morphology remained typically layered, indicating thermal stability [25]. These studies demonstrated that high-entropy design was an effective way to tune the mechanical and thermal properties of MAX phases.

However, current research on high-entropy MAX phases mostly focuses on synthesis processes [26], electromagnetic properties [27] and friction behavior [28,29]. Studies on their damage evolution mechanisms, microstructural strengthening behavior, and service reliability under complex stress conditions remain relatively insufficient. Particularly during frictional contact, the material surface experiences complex environments including periodic shear stress, localized thermal stress, and oxidation-induced embrittlement. Existing studies point out a complex competitive relationship among oxide film formation, interface adhesion, and microcrack propagation, and the lattice distortion introduced by high-entropy design may influence the critical conditions for crack initiation and surface damage evolution [30,31].

However, the research mentioned above highlights that there still gaps that remain to be studied: (1) the sintering parameters and reactants ratio needed to be adjusted methodically for the high purity of high-entropy MAX phases; (2) the reactions during synthesis process of high-entropy MAX phases remained to be clarified systematically; (3) the correlation between frictional damage and failure mechanisms of high-entropy MAX phases over a wide temperature range remained quite limited. In order to remedy these gaps, this study took the quaternary (TiVNbTa)_2_AlC as the target, systematically investigating its crystal and electrical structures from theoretical calculations and exploring its synthesis process, reaction mechanism, and basic mechanical properties from experiments. Through optimizing powder metallurgy and spark plasma sintering (SPS) processes, high-entropy MAX phase (TiVNbTa)_2_AlC bulk with high purity was prepared, and the strengthening mechanism of lattice distortion was also analyzed. Furthermore, within a wide temperature range from room temperature to 400 °C, the friction coefficient, wear rate, and surface oxidation behavior of the material were systematically evaluated, revealing the influence of temperature on interface damage mechanisms and wear mode transitions. By establishing a complete correlation from the synthesis process, microstructure and macroscopic properties, the substantial impacts of high-entropy design on the fundamental mechanical and tribological behavior of MAX phase materials were clarified. The relevant results provided key experimental evidence for evaluating the service performance of such materials under actual working conditions involving mechanical contact and stress concentration.

## 2. Computational and Experimental Details

### 2.1. Computational Methods

Based on the Hume–Rothery criteria, the formation possibility of a single solid solution could be predicted by the atomic difference [32], electronegativity factor [33] and electron concentration [34] by the following equations:

(1) $$\Delta_{r}=\frac{\left|r_{\max}-r_{\min}\right|}{r_{average}}\times 100\%$$

(2) $$\Delta x=\left|x_{A}-x_{B}\right|$$

(3) $$VEC=\frac{\sum C_{i}V_{i}}{\sum C_{i}}$$

where Δ*_r_* represents the atomic difference of elements in the solid solution; *r_max_*, *r_min_* and *r_average_* are the maximum, minimum and average of the atomic radii of elements in the solid solution, respectively; Δ*x* is the electronegativity factor, which can be possessed by the subtraction of the electronegativity of every two elements in the solid solution; *VEC* represents the valence electron concentration; *C_i_* and *V_i_* are atomic ratio and valence electron value of the element i, respectively. The equations above are calculated by data of Ti, V, Nb and Ta elements (shown in Table 1), since they are located on the M-sites simultaneously and form the high-entropy of MAX phases.

According to the Hume–Rothery criteria, the solid solubility will be limited if Δ*_r_* exceeds 15%, because excessive lattice distortion will increase the system energy, thus tending to form intermetallic compounds or other intermediate phases [35]. According to Equation (1), the Δ*_r_* of Ti/V/Nb/Ta elements was 9.07%, less than 15%, which met the critical conditions of solid solution formation and suggested a lower lattice distortion energy. Among Ti/V/Nb/Ta elements, the electronegativity differences between every two elements were 0.09 (Ti/V), 0.06 (Ti/Nb), 0.04 (Ti/Ta), 0.03 (V/Nb), 0.13 (V/Ta) and 0.1 (Nb/Ta), which were all less than 0.4. This indicated that the atomic bonds were mainly metallic bonds, which could thermodynamically inhibit the precipitation of ordered phases (such as intermetallic compounds), and then created reliable conditions to form the solid solutions. The valence electron values of elements shown in Table 1 were similar. As for the Ti/V/Nb/Ta system with equal molar ratio, *VEC* was 4.75, suggesting the compatibility in the electronic structure [36]. All the results shown above met the Hume–Rothery criteria, thermodynamically demonstrating not only the rationality and feasibility of the Ti/V/Nb/Ta system on M-sites but also the feasibility of high-entropy MAX phases (TiVNbTa)_2_AlC.

**Table 1:** The essential information of Ti, V, Nb and Ta elements [37].

Elements	Atomic Radius/Å	Pauling Electronegativity Scale	Valence Electron
Ti	1.47	1.54	4
V	1.34	1.63	5
Nb	1.46	1.6	5
Ta	1.46	1.5	5

**Figure 1:**
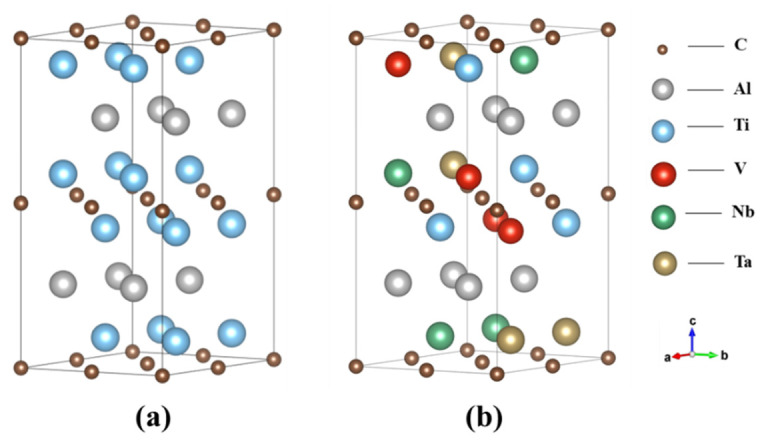
The 2 × 2 × 1 supercell crystal structures of (**a**) Ti_2_AlC; (**b**) (TiVNbTa)_2_AlC.

Nano-laminated MAX phases exhibit the hexagonal structure with P6_3_/mmc (No. 194) symmetry. Figure 1a shows the crystal structure of Ti_2_AlC. The supercell size was 2 × 2 × 1, which contained 32 atoms. In Ti_2_AlC, the Wyckoff locations of Al, C and Ti were (0.33, 0.67, 0.75), (0, 0, 0) and (0.33, 0.67, *z*), respectively. In order to simulate the disordered atomic distribution on M-sites, the HE-MAX (TiVNbTa)_2_AlC structure (shown in Figure 1b) was built based on the SQS (special quasi-random structure) method via ATAT 3.36 (Alloy Theoretic Automated Toolkit, Brown university, Providence, RI, USA). The ratio of elements was Ti at.% = V at.% = Nb at.% = Ta at.% = 25%.

Based on the density functional theory (DFT), the first principles calculations were carried out by Materials Studio 2020 (BIOVIA, Paris, French) in the CASTEP code. The generalized Gradient Approximation (GGA) in the Perdew–Burke–Ernzehof (PBE) framework was used to describe the exchange-correlation energy. To optimize the geometry of crystal structures, the low-memory Broyden–Fletcher–Goldfarb–Shannon method (LBFGS) was utilized. In this work, the convergence tolerance was 5.0 × 10^−6^ eV/atom for the total energy [38], the maximum ionic displacement was 5.0 × 10^−4^ Å [39], and the maximum stress was 0.02 GPa with 0.01 eV/Å in the Hellmann–Feynman force [40]. The cut-off energy was 500 eV with a K-point 7 × 7 × 3 grid for the Brillouin zone [41].

### 2.2. Fabrication Process

Elemental powders, consisting of titanium (99.9% purity, ~48 μm), vanadium (99.7%, ~48 μm), niobium (99.95%, ~38 μm), tantalum (99.9%, ~48 μm), aluminum (99%, ~48 μm), and carbon (99.95%, 18~20 μm), were used to fabricate HE-MAX (TiVNbTa)_2_AlC. The materials were weighed at molar ratios of M:Al:C = 2:1.2:(0.6~1.1) (signed as 0.6C~1.1C, respectively) with the excessive Al to compensate for its evaporation loss [42]. The powder mixture was thoroughly mixed by passing through a 200-mesh sieve and subsequently sintered pressurelessly at 1550 °C for 2 h. Then, according to this optimal ratio, reactant powders were sintered at 1250~1550 °C for 2 h to find the optimal temperature for preparing (TiVNbTa)_2_AlC. Additionally, the raw powders were sintered at 750~1150 °C to study the phase transformation process. The sintered products were ground into powders and then sintered into dense blocks using SPS at 1200 °C and 30 MPa for further tests.

### 2.3. Characterization

X-ray diffractometry (XRD, D8 ADVANCE, Bruker, Karlsruhe, Germany) with Cu Kα radiation was employed for the phase compositions. The mass fraction was approximated by the K-value method [43]:

(4) $$W_{i}=\frac{I_{i}/K_{i}}{\sum_{i=1}^{n}I_{i}/K_{i}}\times 100\%$$

where $W_{i}$, $I_{i}$ and $K_{i}$ were the mass fraction, the intensity of the highest peak and the reference intensity of the i phase, respectively. The surface microstructure of sintered samples was observed using a scanning electron microscope (SEM, S4800, Hitachi, Tokyo, Japan) at the acceleration voltage of 20 kV and the vacuum regime 3 × 10^−3^ Pa with an energy-dispersive spectrometer (EDS, Aztec UltimMax 100, Oxford Instruments, Abingdon, UK). A microhardness tester (MH-500D, Everone Instrument, Shanghai, China) was utilized to measure the Vickers hardness of samples at a load of 100 kgf and a dwell time of 15 s [44]. The measurements were employed 14 times, and the average value was taken after deleting the items with large deviations. A heatable friction and wear tester was employed for reverse grinding between samples and a G25 bearing steel ball (Φ5 mm) for the real-time detection of friction force and coefficient of friction (COF) at R.T. to 400 °C. A load of 4 N was applied perpendicular to the test surface. Each experimental condition was repeated three times to obtain the averages. The wear rate was defined by Equation (5):

(5) $$W_{R}=\frac{wt}{2Fn}$$

where $W_{R}$, w, t, F and n were the wear rate, scar width, wear thickness, load, and the rotation cycles calculated by *n* = rotational speed (200 rpm) × test time (≥40 min).

## 3. Results and Discussion

### 3.1. Structural and Mechanical Properties of HE-MAX (TiVNbTa)_2_AlC from the First Principles Calculations

After the geometric optimization, the lattice parameters are listed in Table 2. With the comparison, lattice parameters of Ti_2_AlC in this work agreed with other reported studies, suggesting the rationality and accuracy of the calculated parameters and the constructed supercells. As for the HE-MAX (TiVNbTa)_2_AlC, the a-lattice was slightly smaller than that of Ti_2_AlC, while the c-lattice parameter had few significant changes. The V atom was smaller than Ti, thus causing declination of lattice parameters.

**Figure 2:**
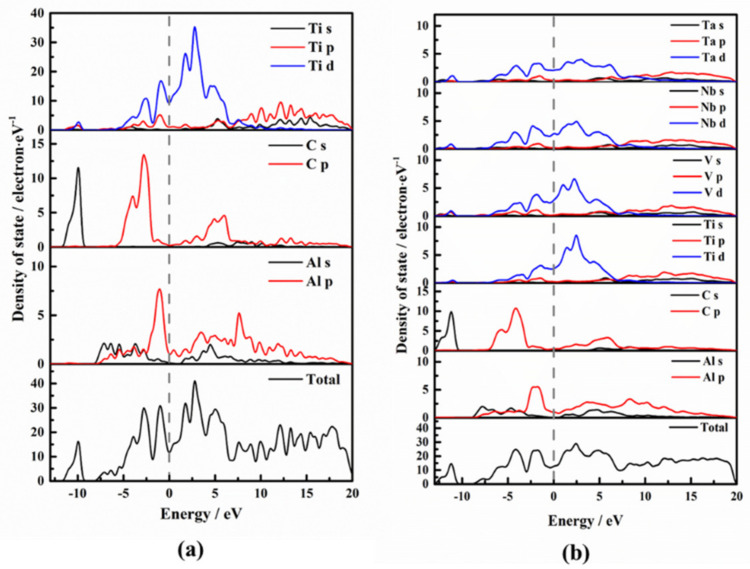
TDOS and PDOS curves of (**a**) Ti_2_AlC and (**b**) (TiVNbTa)_2_AlC.

Properties of the materials are fundamentally determined by their electronic structure and atomic bonds. Figure 2 shows the comparison of TDOS (total density of states) and PDOS (partial density of states) between Ti_2_AlC and HE-MAX (TiVNbTa)_2_AlC. The Fermi level, plotted as the dashed line at zero energy, approximately represented the highest energy level occupied by electrons at the temperature in the system [48]. In Figure 2a, the TDOS value of Ti_2_AlC was over zero at the Fermi level, suggesting metallic conductivity. In the PDOS, it could be seen that the hybridization between Ti 3d electrons and Al 2p electrons contributed to the electrical conductivity of Ti_2_AlC at the Fermi level. The Ti 3d electrons hybridized with C 2s/ 2p electrons, indicating the strong covalent bonding between Ti and C atoms, while the shape of Al 2p electrons overlapped partially with that of Ti 3d electrons, suggesting the weaker hybridization between the two states. Meanwhile, the hybridization between Ti and C atoms was stronger in the lower energy range than that between Ti and Al atoms, suggesting that the Ti-C bonds were stronger than Ti-Al bonds. Ti_2_AlC simultaneously possessed both metallic and covalent bonds, which explained, from the electrical structure, why MAX phases exhibited both metallic-like and ceramic-like properties.

In Figure 2b, the electrical structure of (TiVNbTa)_2_AlC was similar to that of Ti_2_AlC. After doping the Nb, V and Ta elements, the value of TDOS curves of (TiVNbTa)_2_AlC at the Fermi level was higher than that of Ti_2_AlC, possibly predicting higher metallic conductivity. This could be explained by the contribution of d electrons of designed elements on the M-sites. Meanwhile, the peak distribution of elements also had a significant change. The PDOS peak of the C 2s orbit moved to a lower energy range from −9.93 eV to −11.16 eV, due to the contribution of more d electrons provided by the M-site alloying and the strength increment in M-C covalent bonds. The peak of the Al 2p orbit centered at −1.07 eV in Ti_2_AlC, while it broadened and exhibited a left shift after M-site alloying.

This phenomenon could be explained from two aspects: (1) The lattice distortion caused by partially substituting Ti atoms changed the atomic layer spacings of (TiVNbTa)_2_AlC, subsequently squeezing Al atoms. Since the spindle-shaped p orbit was highly sensitive to the positions of neighbored atom, the substitution of Nb, V and Ta intensified the superposition of Al 2p orbit and the d orbits of M-site atoms, which reduced the electron concentration and broadened the peaks when projected onto the PDOS curves. (2) After M-site alloying, Ti 3d, V 3d, Nb 4d and Ta 5d orbits jointly hybridized with the Al 2p orbit, which enhanced the hybridization of the Al 2p orbit and the d orbits of M-site atoms and therefore widened peaks of the Al 2p orbit in PDOS curves.

**Figure 3:**
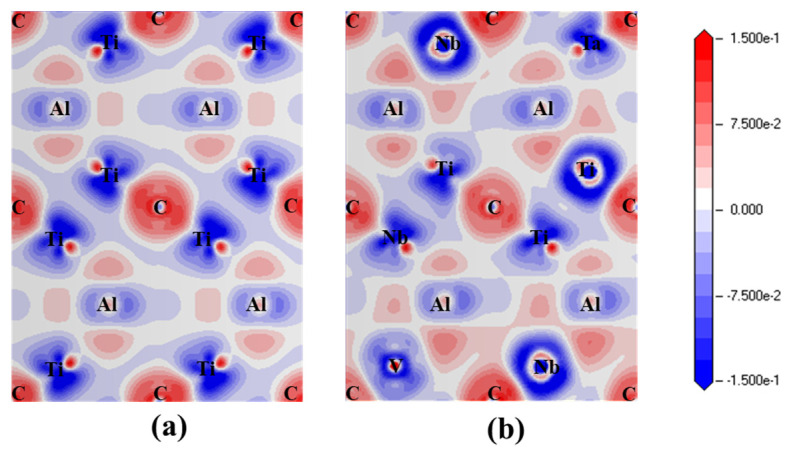
Charge density differences of (**a**) Ti_2_AlC and (**b**) (TiVNbTa)_2_AlC parallel to the (110) plane.

The charge density differences were utilized to reveal the changes in chemical bonds brought by M-site alloying. Considering the Wyckoff positions of atoms, distributions of Ti_2_AlC and (TiVNbTa)_2_AlC parallel to the (110) crystal plane were used for observation, as shown in Figure 3. The blue regions represented the loss of charges, while the red regions represented the gain of charges. In Ti_2_AlC in Figure 3a, the superposition of electron clouds of Ti and C atoms and the directional charge density differences (in which Ti atoms lost charges and C atoms gained charges) indicated the formation of the Ti-C covalent bonds. The positive charge density between Ti and Al atoms, along with no distinguished directions, suggested that Ti and Al atoms were bonded by metallic bonds. Ti_2_AlC simultaneously possessed Ti-C covalent bonds and Ti-Al metallic bonds, thereby exhibiting both ceramic-like (e.g., high strength and hardness and large modulus) and metallic-like properties (e.g., thermal and electrical conductivity and good machinability). In Figure 3b, after adding V, Nb and Ta atoms, the value of charge density between M-site atoms and C atoms significantly increased, indicating the intensification of charge transfer between them and the corresponding enhancement of M-C bonds. Meanwhile, the increasing superposition of electron clouds between M-site atoms and Al atoms showed the incremental interaction between them and the subsequent strengthening of M- Al bonds, which was consistent with the conclusions of DOS curves.

Elastic constants, one of the most important parameters to evaluate mechanical properties, are critical physical parameters to describe the deformation behaviors under load, which could be calculated via the stress–strain method. Since MAX phases belonged to the hexagonal structures, the elastic constants contained five independent variables (*C*_11_, *C*_12_, *C*_13_, *C*_33_ and *C*_44_) [49]. Table 3 lists the calculated elastic constants of Ti_2_AlC and (TiVNbTa)_2_AlC. The elastic constants of optimized Ti_2_AlC in this work were consistent with the literature. The stability of hexagonal structures could be evaluated according to the Born–Huang stability criteria shown in Equations (6)–(9). After the calculation, Ti_2_AlC and (TiVNbTa)_2_AlC were both mechanically stable.

(6) $$C_{11}>0$$

(7) $$C_{11}-C_{12}>0$$

(8) $$C_{44}>0$$

(9) $$\left(C_{11}+C_{12}\right)\times C_{33}-2C_{13}^{2}>0$$

*C*_11_ and *C*_33_ corresponded to the longitudinal elastic constants along the a- and c-axes, respectively, representing the ability to withstand uniaxial compression [50]. *C*_12_ and *C*_13_ referred to the shear stress along the b- and c-axes when materials were loaded along the a-axis. In Table 3, *C*_11_ was larger than *C*_33_ in both Ti_2_AlC and (TiVNbTa)_2_AlC, which indicated that they both had the largest compressive resistance along the a-axis. As V, Nb and Ta elements doped on the M-sites, the value of *C*_11_, *C*_12_ and *C*_13_ significantly increased, suggesting the increasing deformation resistance under the a-axis compression, that is to say, the deformation resistance of Ti_2_AlC could be enhanced by M-site alloying. Moreover, *C*_44_ and *C*_66_ referred to the shear resistance along the b- and c-axes, respectively [51,52]. The calculated values of *C*_44_ and *C*_66_ were not equal, whether in Ti_2_AlC or in (TiVNbTa)_2_AlC, indicating the anisotropy of shear modulus in MAX phases.

**Table 3:** Elastic constants of Ti_2_AlC and (TiVNbTa)_2_AlC (units: GPa).

	*C*_11_	*C*_12_	*C*_13_	*C*_33_	*C*_44_	*C*_66_	Ref.
Ti_2_AlC	302.1	62.1	59.9	265.2	109.8	120.0	This work
	302.0	68.0	64.0	268.0	107.0		[53]
	304.0	60.1	58.0	271.1	110.8		[49]
(TiVNbTa)_2_AlC	321.1	96.7	95.0	317.5	148.6	112.2	This work

When materials deformed in the elastic stage, there was a linear relationship between stress and strain with the linear parameters containing bulk modulus *B*, elastic modulus *E* and shear modulus *G*, which could be calculated by Equations (10)–(12), respectively [54,55]

(10) $$B=(B_{V}+B_{R})/2$$

(11) $$G=(G_{V}+G_{R})/2$$

(12) E=9BG/(3B+G)

where *B_V_* and *B_G_* were the bulk modulus defined by the Voigt and Reuss approximations, respectively, as well as *G_V_* and *G_R_*. The bulk modulus *B* reflected the ability to resist changes in volume when the material was compressed. The larger its value, the more difficult it was to compress materials. The elastic modulus *E*, the key index to evaluate the uniaxial stiffness of materials, describes the linear relationship between stress and strain in the elastic stage when materials are compressed or tensioned uniaxially. The shear modulus *G* was the linear parameter between shear stress and the corresponding shear strain, serving as the measurement for the shear deformation resistance. The Poisson’s ratio *ν* was the ratio of the transverse strain to the axial strain when the materials were uniaxial loaded, reflecting the ability to maintain dimensional stability in the transverse direction. As shown in Table 4, as V, Nb and Ta atoms doped on the M-sites, the values of *B*, *E*, *G* and *ν* significantly increased, indicating the increment of stiffness and deformation resistance.

Moreover, the ductile-brittle behavior could also be predicted according to the Pugh, Poisson and Pettifor criteria. In the Pugh criterion [56], the ratio of *B*/*G* was utilized to judge the ductility or brittleness of materials. When *B*/*G* > 1.75, the material exhibited ductile behavior, with higher *B*/*G* values indicating greater ductility. When *B*/*G* < 1.75, the material exhibited brittle behavior. In the Poisson criterion [57], which relied on the Poisson’s ratio of materials, when *ν* > 0.26, the material behaved as ductile; when *ν* < 0.26, the material behaved as brittle. The Pettifor criterion [58] used Cauchy pressure (*C*_12_–*C*_44_) to classify the ductility or brittleness: positive Cauchy pressure indicated ductile behavior, while negative Cauchy pressure suggested brittle behavior. Table 5 shows the *B*/*G* ratio, Cauchy pressure and Poisson’s ratio of Ti_2_AlC and (TiVNbTa)_2_AlC. Compared to traditional ceramics, MAX phases exhibited higher *B*/*G* ratios, indicating superior ductility, which resulted from the unique nano-layered structure. Once upon the impact, the layered structure effectively dissipated energy, prevented the crack propagation and thereby contributed to the well impact resistance. Ti_2_AlC and (TiVNbTa)_2_AlC were both classified as brittle materials according to the three criteria above. It could be seen that the *B*/*G* ratio and the Poisson’s ratio increased after M-site alloying, suggesting a decline in brittleness. According to the Pettifor criterion, HE-MAX (TiVNb)_2_AlC exhibited little higher brittleness than Ti_2_AlC, which was different from the conclusions based on the Pugh and Poisson criteria. This inconsistency of brittleness conclusions indicated that the inherent brittleness of (TiVNb)_2_AlC was not low enough [57]. In order to make sure the brittleness tendency after M-site alloying, other calculations were listed in Table 5 and further analyzed. The Cauchy pressure was the key parameter to evaluate not only the ductility or brittleness, but also the nature of chemical bonding [59]. A negative Cauchy pressure also indicated the strong covalent bond characteristics. The Cauchy pressure of (TiVNb)_2_AlC was more negative than that of Ti_2_AlC, which indicated that the two MAX phases were both dominated by directional covalent bonding, and the covalency increased after M-site alloying.

In addition, the fracture toughness *K_IC_* [60] was calculated following Equation (13) to estimate the crack extension resistance. The brittle index *M* [61] calculated by Equation (14) could be used to describe the damage tolerance, with smaller *M* indicating better damage tolerance.

(13) $$K_{IC}=V_{0}^{1/6}G{\left(\frac{B}{G}\right)}^{1/2}$$

(14) $$M=\frac{H_{V}}{K_{IC}}$$

where *V*_0_ represented the volume of the crystals shown in Table 2; *B* and *G* referred to the bulk and shear modulus, respectively. In Table 5, the *K_IC_* of he-max was much higher than Ti_2_AlC, forecasting that (TiVNbTa)_2_AlC might be better in the crack extension resistance than Ti_2_AlC. Additionally, the brittleness index *M* of (TiVNbTa)_2_AlC was smaller than that of Ti_2_AlC, predicting the perhaps better damage tolerance of (TiVNbTa)_2_AlC. After comprehensive evaluations of items shown in Table 5, it could be forecasted that the enhancement of the brittleness, the crack extension resistance and damage tolerance of (TiVNbTa)_2_AlC could be achieved by M-site alloying.

Since in MAX phases M_6_X layers and A layers are arranged alternately along the c-axis, the unique layered structure and high lattice parameter ratio of c/a contributed to the strong anisotropy, which was also reflected in their elastic anisotropy. Elastic anisotropy included the general anisotropy index *A_U_*, compression anisotropy *A_B_*, and the shear anisotropy *A_G_*, which could be calculated according to Equations (15)–(17):

(15) $$A_{U}=5G_{V}\;/G_{R}+B_{V}\;/B_{R}-6\;$$

(16) $$A_{G}={(G}_{V}-G_{R})/{(G}_{V}+G_{R})\;$$

(17) $$A_{B}={(B}_{V}-B_{R})/{(B}_{V}+B_{R})$$

where *B_V_* and *B_R_* were the modulus approximation using Voigt and Reuss function simulations, respectively, as well as *G_V_* and *G_R_*. Table 6 lists the anisotropy constants of Ti_2_AlC and (TiVNbTa)_2_AlC. The strong anisotropy could be observed in both Ti_2_AlC and (TiVNbTa)_2_AlC. After M-site alloying, the values of *A_U_*, *A_B_* and *A_G_* all rose, which could be explained by the fact that M-site alloying enhanced the inhomogeneity of the atomic arrangement in crystal cells.

### 3.2. Process Parameters of (TiVNbTa)_2_AlC

**Figure 4:**
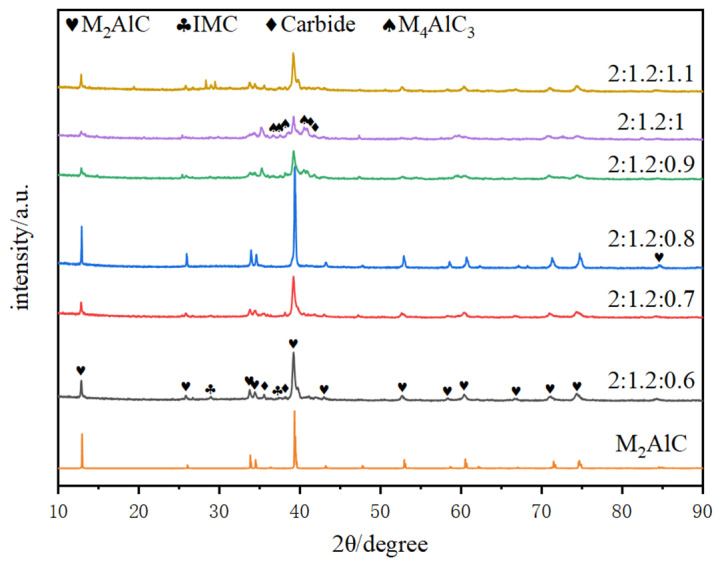
XRD patterns of sintered products by different raw materials compositions. (TiC-PDF#97-009-3504; (TaTi)_2_C-PDF#97-007-7408; Nb_2_Al-PDF#04-007-1667; Ta_5_Al_3_-PDF#04-001-3318; V_2_C-PDF#04-001-8724; Ta_2_C-PDF#97-004-0955; M_2_AlC: XRD pattern was calculated from the crystal structure shown in Figure 1b).

Figure 4 shows the XRD results of sintered products with different reactant ratios. The XRD pattern of Sample 0.6C mainly showed peaks of M_2_AlC, accompanied by many characteristic peaks of intermetallic compounds (IMCs) and carbides (MC), indicating low purity of the M_2_AlC phase formed at 1450 °C with this ratio. The M_2_AlC peaks in Sample 0.7C strengthened, and there were almost no IMC characteristic peaks, indicating improved purity of (TiVNbTa)_2_AlC [62]. The XRD pattern in Sample 0.8C of the product almost entirely consisted of characteristic peaks of M_2_AlC, indicating high purity of the M_2_AlC phase formed at 1550 °C with this ratio. As the C ratio was up to 0.9, the intensity of MC peaks significantly rose, and characteristic peaks of M_4_AlC_3_ appeared, indicating a significant increase in impurity content and a decrease in M_2_AlC purity. This suggested that excess carbon promoted preferential carbide formation, inhibiting stable MAX phase growth. It indicated a narrow compositional window for high-entropy MAX phase formation, essentially relating to element diffusion kinetics and local chemical equilibrium in the multi-component system. In Sample 1C, the M_2_AlC characteristic peaks weakened while M_4_AlC_3_ and MC peak intensities increased, indicating relatively low purity of M_2_AlC under this condition. In sample 1.1C, the M_2_AlC peak intensity apparently receded, while characteristic peak intensities of M_4_AlC_3_ and MC rose notably, showing the following purity reduction in M_2_AlC. Therefore, the ratio M:Al:C = 2:1.2:0.8 yielded relatively pure M_2_AlC.

**Figure 5:**
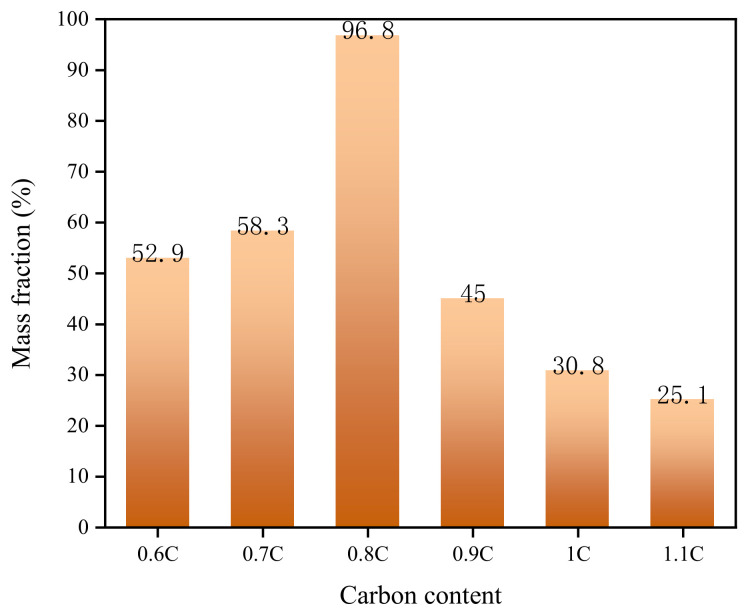
Mass fraction of (TiVNbTa)_2_AlC in sintered products.

The mass fractions of (TiVNbTa)_2_AlC from the K-value method are shown in Figure 5. With the increasing C ratio, the mass fraction of (TiVNbTa)_2_AlC showed an inverted “V” tendency, which reached the peak at 0.8C. Therefore, the 0.8C ratio fabricated (TiVNbTa)_2_AlC with the highest purity.

**Figure 6:**
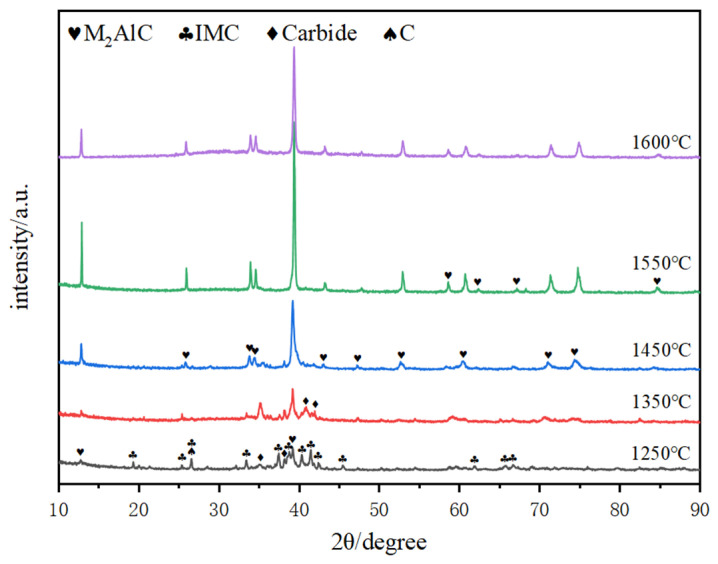
XRD patterns of samples at different temperatures. (TiAl-PDF#04-006-6741; NbC-PDF#04-001-1554; Nb_2_Al-PDF#04-007-1667; Nb_3_Al-PDF#04-001-3143; TiC-PDF#97-009-3504; Ta_2_C-PDF#97-004-0955; NbAl_3_-PDF#00-004-2210; TaAl_3_-PDF#00-004-2210; M_2_AlC: XRD pattern was calculated from the crystal structure shown in Figure 1b).

The XRD patterns of the products obtained by Sample 0.8C at different temperatures are shown in Figure 6. When the sintering temperature was 1250 °C, M_2_AlC peaks had already shown, but were also accompanied by many IMC and MC impurities, such as Ti-Al and Nb-Al intermetallics, TiC and unreacted carbon, indicating lower purity of sintered products. With the increasing sintering temperature, the intensity of IMC peaks gradually weakened, while that of M_2_AlC peaks rose, indicating more sintered IMC and MC. When the sintering temperature reached 1450 °C, IMC and MC peak intensities significantly weakened, indicating significantly reduced impurity content and increased M_2_AlC purity at 1450 °C. Once the temperature turned to 1550 °C, M_2_AlC peak intensity increased apparently, MC peaks decreased and the XRD pattern showed almost no other impurity peaks, indicating high purity of M_2_AlC. Above this sintering temperature, MC peak intensity increased, indicating MAX phase decomposition into more carbides at high temperatures, limiting the upper sintering temperature.

**Figure 7:**
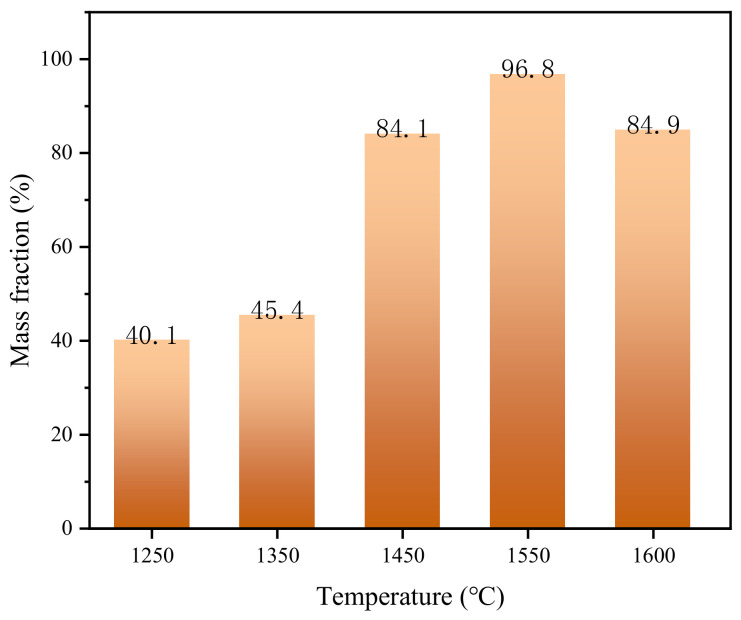
Graph showing the variation in the mass fraction of high-entropy (TiVNbTa)_2_AlC with temperature.

Figure 7 shows the mass fraction of (TiVNbTa)_2_AlC calculated using the K-value method. With the enhanced sintering temperature, the mass of HE-MAX increased first and then declined, with the peak at 1550 °C. Thus, 1550 °C was chosen for the highest purity. In summary, a ratio of M:Al:C = 2:1.2:0.8 and sintering at 1550 °C resulted in M_2_AlC with the highest purity.

Compared with traditional unitary MAX phases, differences in atomic size and diffusion rates among different elements in the high-entropy system significantly affected the phase formation process. On one hand, severe lattice distortion caused by a multi-element solid solution increased the configurational entropy of the system, thereby helping to stabilize the layered MAX structure; on the other hand, the diffusion sluggishness effect reduced the reaction velocity, which made it easier for metastable phases like MC to form in local regions. Therefore, the formation of HE-MAX phases was controlled not only by thermodynamic driving forces but also by complex diffusion kinetics.

### 3.3. Reaction Mechanism of HE-MAX (TiVNbTa)_2_AlC

**Figure 8:**
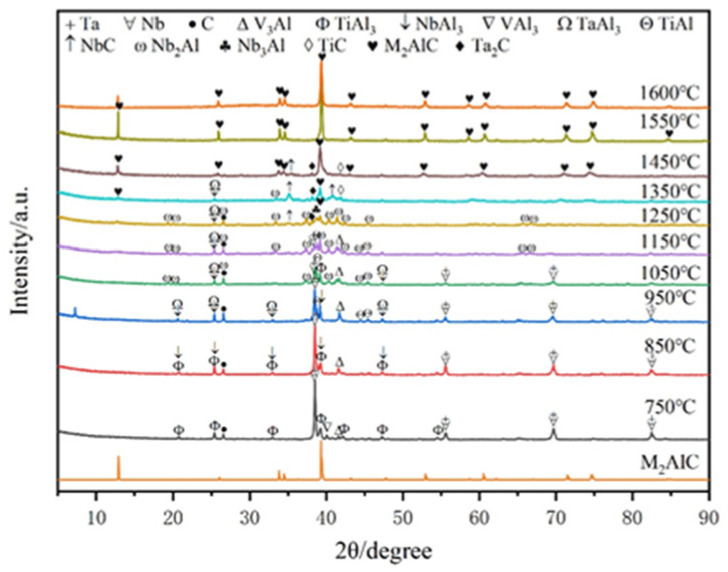
XRD patterns of samples with various temperatures.

To study the reaction path during (TiVNbTa)_2_AlC sintering, XRD patterns of samples with raw material ratio M:Al:C = 2:1.2:0.8 sintered from 750 to 1600 °C are shown in Figure 8 and Table 7.

At 750 °C, molten Al and M-site elements first reacted into intermetallic compounds (IMCs). TiAl_3_, VAl_3_, and V_3_Al were recognized in XRD patterns, indicating the corresponding reaction shown in the following equations:

(18) $$\mathrm{Ti}\;+\;3\mathrm{Al}\;=\;{\mathrm{TiAl}}_{3}$$

(19) $$V\;+\;3\mathrm{Al}\;=\;{\mathrm{VAl}}_{3}$$

(20) $$3V\;+\;\mathrm{Al}\;=\;V_{3}\mathrm{Al}$$

As the temperature further rose, more metals reacted with Al, IMC peak intensities continuously increased, and peaks of individual M metals decreased, indicating more M-site elements participated in the reactions with Al as follows:

(21) $${\mathrm{TiAl}}_{3}\;+\;\mathrm{Ti}\;=\;{\mathrm{TiAl}}_{2}\;+\;\mathrm{TiAl}$$

(22) $${\mathrm{TiAl}}_{3}+{\mathrm{TiAl}}_{2}+3\mathrm{Ti}=5\mathrm{TiAl}$$

(23) $$\mathrm{Ta}+3\mathrm{Al}={\mathrm{TaAl}}_{3}$$

At 1050 °C, Nb_2_Al peaks appeared, indicating transitions occurred in Nb-Al intermetallics:

(24) $$5Nb+{NbAl}_{3}=3{Nb}_{2}Al$$

(25) $$2Nb+Al={Nb}_{2}Al$$

After sintering at 1150 °C, weak carbide Ta_2_C characteristic peaks appeared in the XRD pattern, indicating a reaction between Ta metal powder and C powder:

(26) $$2\mathrm{Ta}\;+\;C\;=\;{Ta}_{2}C$$

When the sintering temperature reached 1250 °C, MC and IMC reacted to form M_2_AlC. The peaks of M-site elements completely disappeared with the observation of various IMCs, MCs, and M_2_AlC. The reactions were as follows:

(27) $${\mathrm{Ti}}_{3}\mathrm{Al}\;+\;\mathrm{TiAl}\;+\;2C\;=\;2{\mathrm{Ti}}_{2}\mathrm{AlC}$$

(28) $${\mathrm{Ti}}_{3}\mathrm{Al}+C={\mathrm{Ti}}_{2}\mathrm{AlC}+\mathrm{TiC}$$

(29) $$\mathrm{TiC}+\mathrm{TiAl}={\;\mathrm{Ti}}_{2}\mathrm{AlC}$$

(30) Nb+C=NbC

(31) $$5\mathrm{NbC}+2{\mathrm{Nb}}_{2}\mathrm{Al}+{\mathrm{NbAl}}_{3}=5{\mathrm{Nb}}_{2}\mathrm{AlC}$$

(32) $$V_{3}\mathrm{Al}+2C=V_{2}\mathrm{AlC}+\mathrm{VC}$$

(33) $${Ta}_{2}\mathrm{Al}+C={\;\mathrm{Ta}}_{2}\mathrm{AlC}$$

In the temperature range of 1250~1450 °C, M_2_AlC peaks strengthened, IMC peaks gradually weakened, and carbide peaks strengthened, suggesting the formation of M_2_AlC, while some MC were accompanied by the formation of M_2_AlC. Additionally, as temperature increased, atomic diffusion activation energy decreased, and element migration rates accelerated, which provided more favorable kinetic conditions for carbide nucleation and subsequently promoted the increasing MC phase content [63]. At 1550 °C, IMCs were totally transformed into M_2_AlC together with a small amount of carbide impurities. At 1600 °C, M_2_AlC partially decomposed into high-entropy carbide MC, correspondingly polluting HE-MAX.

**Figure 9:**
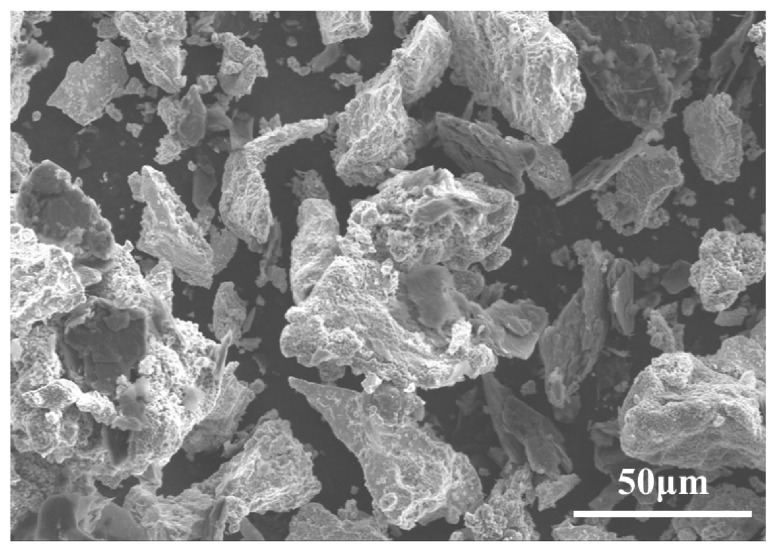
SEM image of the sintered product at 850 °C.

**Figure 10:**
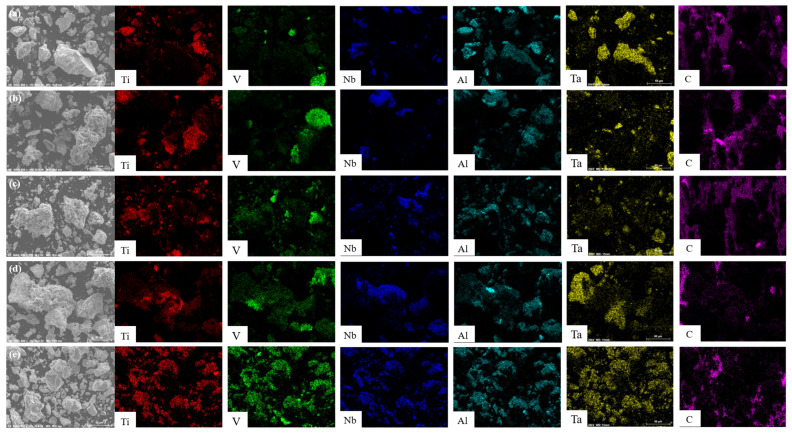
(**a**–**e**) EDS spectra of Sample 0.8C at 1150, 1250, 1350, 1450 and 1550 °C, respectively. Scale bar: 60 μm.

Figure 9 shows the SEM image of Sample 0.8C sintered at 850 °C, while the EDS results of the products sintered at 1150~1550 °C are presented in Figure 10. In Figure 9, the microstructure of the product sintered at 850 °C mainly consisted of Ti-Al- and Nb-Al-rich intermetallic compounds (IMCs) together with residual raw material particles. This indicated that reactions among the constituent elements had just initiated at this relatively low temperature. Ti and Nb were preferentially reacted with molten Al to form Al-rich intermetallics. Up to 1150 °C, a slight carbide (MC) phase occurred in addition to IMC structures. Partial overlap between the C and M element distributions could be observed in the EDS maps in Figure 10a, indicating that reactions between the transition metal elements and carbon had occurred. At 1250 °C, although previous XRD analysis confirmed the formation of M_2_AlC, the characteristic layered morphology of the MAX phase was still difficult to distinguish in the SEM images, and the microstructure remained dominated by IMCs, as shown in Figure 10b. Up to 1450 °C, the amount of carbide structures increased significantly, while the IMC structures nearly disappeared. Meanwhile, the typical laminated morphology of the MAX phase became distinguishable in Figure 10c, indicating that the M elements continuously reacted with carbon to form MC phases along with more intermetallic compounds transforming into M_2_AlC. At 1550 °C, the EDS elemental maps revealed a high degree of overlap among the four M elements in Figure 10d, suggesting a homogeneous solid-solution distribution and a relatively high purity of the high-entropy (TiVNbTa)_2_AlC.

Quantitative EDS results were conducted on the (TiVNbTa)_2_AlC powders obtained by pressureless sintering at different temperatures in Table 8. At 1450 °C, the overall ratio of the solid-solution M-site elements was generally consistent with Ti:V:Nb:Ta = 1:1:1:1, and the ratio of M-site/Al in (TiVNbTa)_2_AlC was also close to the stoichiometry of 2:1. This followingly indicated that the synthesis strategies of both sightly excessive Al to make up for the evaporation loss and the sintering parameters (1450 °C) chosen brought the achievement of HE-MAX (TiVNbTa)_2_AlC with high purity.

### 3.4. Mechanical Properties

Table 9 presents the Vickers hardness of Ti_2_AlC and (TiVNbTa)_2_AlC. The experimentally measured hardness of (TiVNbTa)_2_AlC reached 6.4 GPa, which was higher than the hardness values of the four corresponding ternary MAX phases. The increment in hardness mainly contributed to the severe lattice distortion after M-site alloying. Additionally, differences in diffusion kinetics among multiple elements promoted the growth of a high-density dislocation network. Lattice distortion created local stress fields that impeded dislocation slip, which demanded more energy to surpass the barriers and then raised the material’s resistance to plastic deformation. The pinning effect restrained the grain boundary migration, and the high-density dislocations associated with lattice distortion also significantly contributed to the strengthening and hardening effects, as widely reported in many medium- and high-entropy materials [64].

**Figure 11:**
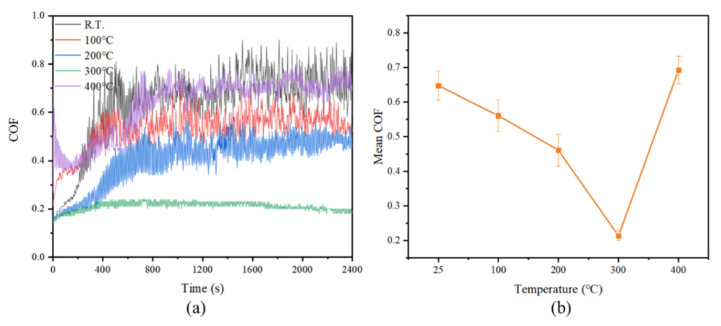
(**a**) Friction coefficient-time curves and (**b**) average friction coefficients of high-entropy (TiVNbTa)_2_AlC at different temperatures.

Figure 11 shows the friction coefficient (COF)-time curves and mean COF (MCOF) values of the high-entropy (TiVNbTa)_2_AlC samples at different testing temperatures. Under R.T. to 100 °C, the COF curves fluctuated strongly, and the MCOF decreased from 0.65 to 0.56. This behavior indicated the dominant adhesive wear between the sample and the bearing counterpart. During the friction, adhesive junctions formed and sheared off repeatedly. At the initial heating stage, stable oxide films had not yet formed on the surface. The repeated adhesion and the shear fracture at local contact points resulted in the continuous fluctuations in the contact stress.

**Figure 12:**
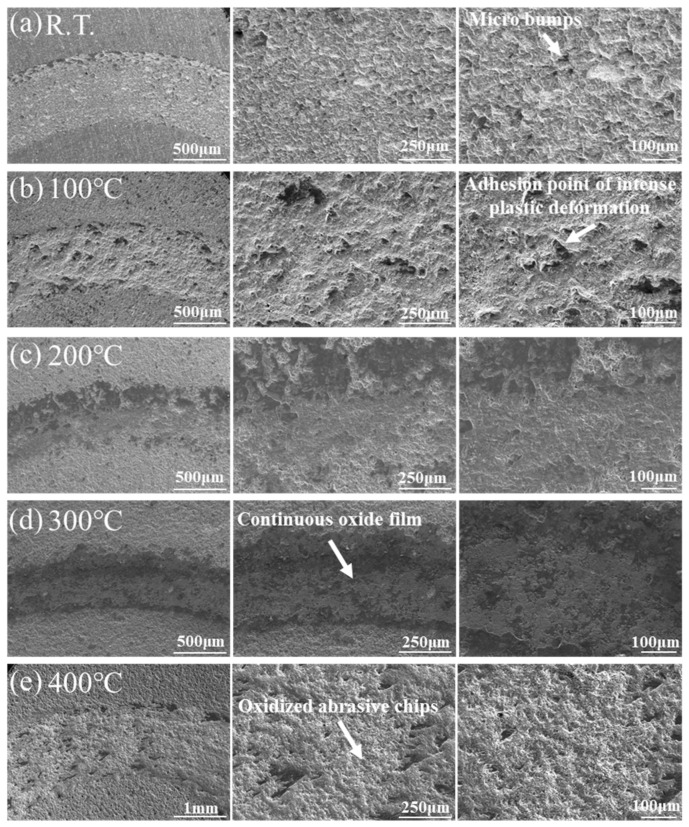
Surface morphologies of wear tracks of high-entropy (TiVNbTa)_2_AlC after friction tests at different temperatures.

At 200~300 °C, the MCOF was further reduced to approximately 0.2, while the fluctuation amplitude became significantly smaller. This indicated that increasing temperature promoted the formation of oxide films on the surface. In Figure 12c,d, the formation of oxide layers with discontinuous lubrication at the sliding interfaces prevented the real contact between friction pairs. The reduction in friction caused by the oxide films outweighed the increase in friction resistance associated with adhesion, welding and shear fracture, resulting in a continuous decrease in the MCOF.

However, up to 400 °C, COF curves became more fluctuated, and the MCOF suddenly increased. This behavior could be caused by the accelerated oxidation reaction at the increasing temperatures. Although continuous oxide layers theoretically provided lubrication and reduced friction, excessively thick oxide films became brittle and prone to spall during sliding, thus generating abrasive debris and increasing the probability of three-body wear. In addition, the elevated temperature decreased the yield strength of samples, which led to severe plastic deformation in the contact regions. Simultaneously, the interfacial adhesion and micro-welding effects were intensified, correspondingly causing a rougher friction surface as shown in Figure 12e. As a result, the friction coefficient increased rather than decreased.

**Figure 13:**
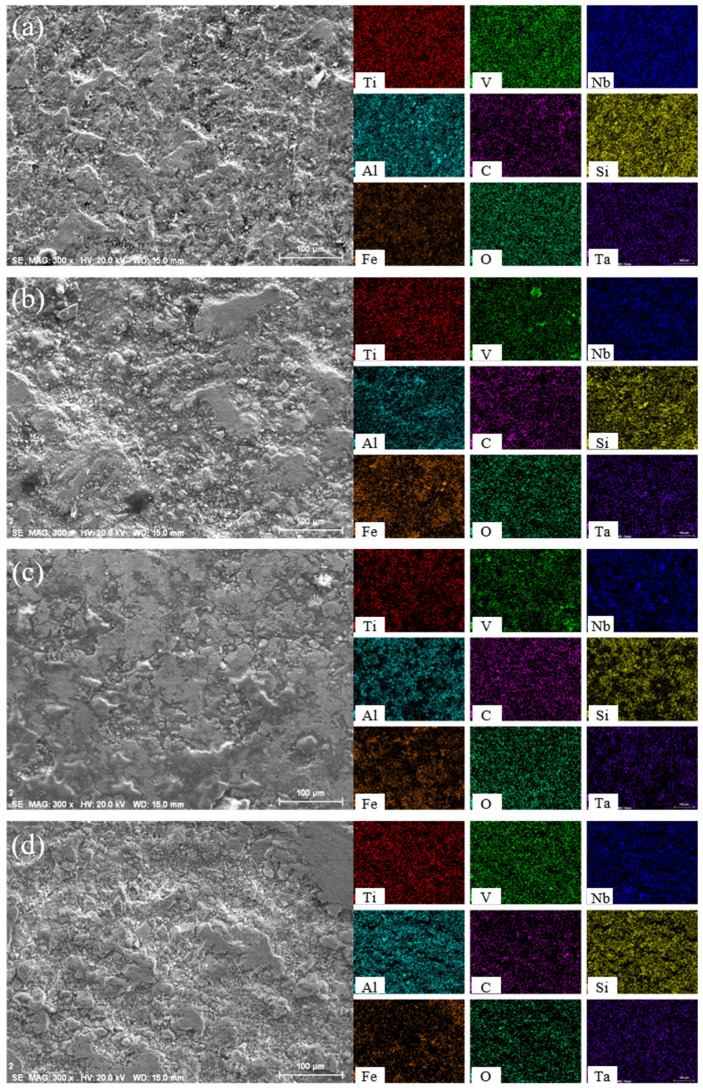
EDS elemental mapping images of the wear tracks of high-entropy (TiVNbTa)_2_AlC after friction tests at (**a**) R.T., (**b**) 200 °C, (**c**) 300 °C, and (**d**) 400 °C.

Figure 13 shows the EDS elemental mapping results of the wear tracks of high-entropy (TiVNbTa)_2_AlC at different temperatures. At R.T., the weak oxygen signal on the wear track is sparsely distributed, indicating that no continuous friction-induced oxide film formed on the worn surface. Under this condition, friction was mainly dominated by direct material contact and shear deformation, resulting in a relatively high friction coefficient. The enrichment of Fe could be detected in the asperity regions, suggesting the material transfer from the steel counterpart to the (TiVNbTa)_2_AlC surface during sliding and then confirming the occurrence of adhesive wear. When the temperature increased to 200 °C, the oxygen signal became significantly stronger and more uniformly distributed across the wear track, implying the creation of relatively intact surface oxide films. The friction-induced oxide layer reduced the interfacial shear force, thereby lowering the friction coefficient. At 300 °C, the oxygen signal became even more pronounced and exhibited the most uniform and dense distribution. The wear track was covered by a relatively complete and stable oxide film. Simultaneously, the distributions of Ta and V overlapped with that of oxygen, indicating the formation of oxide phases mainly composed of Ta_2_O_5_ and V_2_O_5_. These oxide phases exhibited certain lubricating effects and could reduce friction resistance, resulting in excellent tribological performance at this temperature. In addition, the continuous oxide film could hinder surface crack propagation to some extent, thereby improving the stability of surface damage. Up to 400 °C, the oxygen signal weakened and became nonuniform. This indicated that although the oxide layer thickened rapidly at high temperature, its brittleness also increased significantly. Under cyclic shear loading, the oxide film tended to spall and generate abrasive debris. Furthermore, the elevated temperature reduced the local yield strength in samples, resulting in more severe plastic deformation during contact.

The surface profiles of the wear tracks generated on the (TiVNbTa)_2_AlC phase at different temperatures were characterized using a surface profilometer, which is summarized in Table 10. Here, *R_a_* represented the arithmetic mean deviation of the profile, indicating the absolute average height of the surface; *R_q_* was the root-mean-square deviation of the profile and equivalent to the standard deviation of surface height; *R_z_* was the maximum of height difference, and defined as the vertical distance between the highest peak and the lowest valley of the profile; *R_v_* was the maximum of valley depth, representing the distance from the lowest point of the profile to the baseline. *R_z_*_1*max*_ denoted the maximum of the height difference within a single sampling length. The wear track exhibited the lowest surface roughness of samples at 300 °C, while the wear tracks became rougher at other temperatures, which was consistent with the variation in the friction coefficient. The evolution of the wear track morphology could be attributed to the influence of temperatures on the oxide film formation, the local plastic deformation, and the adhesion-induced welding at the contact interface.

**Figure 14:**
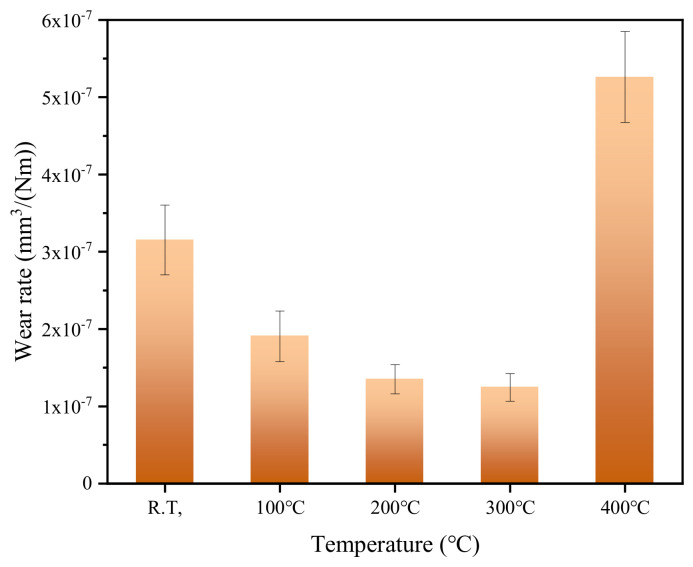
Wear rate of (TiVNbTa)_2_AlC samples at different temperatures.

The wear rates of high-entropy (TiVNbTa)_2_AlC within the temperature range R.T.~400 °C are calculated in Figure 14. The wear rate reduced with the increasing experimental temperature, starting from 3.15 × 10^−7^ mm^3^/(Nm) at room temperature. This indicated that the increasing temperature reduced the strength of asperity contact regions and increased the degree of plastic deformation, thereby aggravating the adhesive wear. In addition, the slight oxidation at this stage was too little for the effectively lubricated film, resulting in severe wear. Subsequently, the wear rate gradually decreased to 1.24 × 10^−7^ mm^3^/(N·m) at 300 °C. At 400 °C, the wear rate rapidly increased again to approximately 5.26 × 10^−7^ mm^3^/(N·m). Except for the behavior observed at 100 °C, the wear rate tended to vary in line with COFs.

Compared with conventional MAX phases Ti_2_AlC [69], V_2_AlC [70], Nb_2_AlC [71], and Ta_2_AlC [69], high-entropy (TiVNbTa)_2_AlC maintained the relatively stable wear behavior over a wider temperature range, thereby reducing the sensitivity of the tribological response to temperature variations. This behavior was closely related to the combined effect of severe lattice distortion with multicomponent oxidation in high-entropy systems. On one hand, lattice distortion increased the resistance to dislocation motion, correspondingly reducing the accumulation of cyclic plastic deformation in the contact surface and alleviating local strain concentration. On the other hand, multiple transition metal elements participated in the formation of composite oxide films during friction, which helped stabilize interfacial shear stress and reduce adhesive damage. Under the cyclic shear loading, the surface layer continuously underwent a dynamic damage evolution involving plastic deformation, microcrack initiation, crack propagation, and material spallation. Owing to its higher hardness and more homogeneous solid-solution structure, HE-MAX (TiVNbTa)_2_AlC could inhibit the formation of surface microcracks and reduce the driving force for crack propagation to some extent. Therefore, the lower wear rate not only reflected improved tribological performance but also demonstrated the beneficial role of high-entropy design in enhancing resistance to surface damage.

## 4. Conclusions

High-entropy (TiVNbTa)_2_AlC MAX phase materials were successfully fabricated by combining pressureless sintering and spark plasma sintering. The crystal and electrical structures were calculated based on DFT calculations, and the reaction process, microstructures, mechanical properties and tribological behavior were systematically investigated. The main conclusions were summarized as follows:

(1) M-site alloying made exhibit stronger bonds and higher elastic properties. Due to the higher value of DOS curves at the Fermi level, (TiVNbTa)_2_AlC possessed better metallic conductivity. The more intense charge transfer between both M-C atoms and M-Al atoms strengthened the M-C and M-Al bonds, respectively. The increased elastic constants and moduli suggested the better deformation resistance of HE-MAX. Moreover, (TiVNbTa)_2_AlC exhibited lower brittleness, better crack extension resistance and damage tolerance, and larger anisotropy than Ti_2_AlC.
(2) The reactant ratio was optimized systematically, and the synthesis reactions were further explored. In the temperature range of 750–1150 °C, the system mainly formed intermetallic compounds such as TiAl_3_, NbAl_3_, and VAl_3_. With further increase in temperature, the IMC and MC phases gradually reacted to form the M_2_AlC phase. The optimization sintering parameters of (TiVNbTa)_2_AlC were 1550 °C with the reactant ratio M:Al:C = 2:1.2:0.8.
(3) The mechanical properties of HE-MAX were subsequently studied. The solid solution strengthening effect mainly originated from severe lattice distortion and sluggish diffusion induced by multiple elements on the M-sites. (TiVNbTa)_2_AlC exhibited pronounced temperature-dependent tribological behavior within the range R.T.~400 °C. At low temperatures, interfacial adhesion and local shear deformation dominated the wear behavior, whereas at higher temperatures, brittle spallation of oxide films and three-body wear gradually became dominant. In addition, compared with conventional MAX phases, high-entropy (TiVNbTa)_2_AlC exhibited superior resistance to surface damage under cyclic contact conditions due to its higher hardness, more homogeneous solid-solution structure, and stable composite oxide films.

## Figures and Tables

**Table 2:** The lattice parameters, cell volume and density of Ti_2_AlC and HE-MAX (TiVNbTa)_2_AlC.

Crystal	a (Å)	c (Å)	Cell Volume (Å^3^)	Density (g/cm^3^)	Ref.
Ti_2_AlC	3.066	13.752	111.8	4.00	this work
	3.066	13.702	111.6		[41]
	3.068	13.752			[45]
	3.065	13.710	111.6		[46]
	3.068	13.716			[47]
(TiVNbTa)_2_AlC	3.058	13.755	111.4	6.72	this work

**Table 4:** Elastic modulus *E*, bulk modulus *B*, shear modulus *G* and Poisson’s ratio ν of Ti_2_AlC and (TiVNbTa)_2_AlC (units: GPa (expect Poisson’s ratio *ν*)).

	*B_V_*	*B_R_*	*G_V_*	*G_R_*	*B*	*G*	*E*	*ν*
Ti_2_AlC	137.02	136.50	113.74	113.42	136.76	113.58	266.87	0.175
(TiVNbTa)_2_AlC	170.32	170.31	126.74	124.36	170.32	125.55	302.35	0.204

**Table 5:** Calculated *B*/*G*, Poisson’s ratio *ν*, Cauchy pressure (*C*_12_–*C*_44_), fracture toughness *K_IC_* and brittleness index *M* for Ti_2_AlC and (TiVNbTa)_2_AlC.

	*B*/*G*	*ν*	*C*_12_–*C*_44_ /GPa	*K_IC_*/MPa × m^0.5^	*M*/ μm^−0.5^
Ti_2_AlC	1.2041	0.175	−47.7	2.73	8.27
(TiVNbTa)_2_AlC	1.2224	0.204	−51.9	3.20	6.43

**Table 6:** The calculations of *A_U_*, *A_B_* and *A_G_* of Ti_2_AlC and (TiVNbTa)_2_AlC.

	*A_U_*	*A_G_*	*A_B_*
Ti_2_AlC	0.0177	0.00139	0.00190
(TiVNbTa)_2_AlC	0.0956	0.00946	2.71 × 10^−5^

**Table 7:** Phase composition during sintering process of Sample 0.8C.

Temperature/°C	Phase Composition
R.T.	Ti, V, Nb, Ta, Al, C
750	Ta, Nb, C, V_3_Al, TiAl_3_, VAl_3_
850	Ta, Nb, C, V_3_Al, NbAl_3_, TaAl_3_, TiAl
950	Ta, Nb, C, V_3_Al, NbAl_3_, TaAl_3_, TiAl
1050	Nb, C, TiAl, Nb_2_Al, NbAl_3_, TaAl_3_, V_3_Al
1150	Nb, C, TiAl, Nb_2_Al, NbAl_3_, TaAl_3_, V_3_Al, MC
1250	C, Nb_2_Al, NbAl_3_, TaAl_3_, Ta_2_C, M_2_AlC, MC
1350	Nb_2_Al, NbAl_3_, TaAl_3_, M_2_AlC, MC
1450	MC, M_2_AlC
1550	MC, M_2_AlC
1600	MC, M_2_AlC

**Table 8:** EDS analysis of sintered (TiVNbTa)_2_AlC powders prepared at different temperatures of Sample 0.8C (units: %).

Temperature	Ti	V	Nb	Ta	Al	C
1150 °C	1.74	0.40	12.17	0.12	28.87	56.70
1250 °C	12.35	0.17	10.25	11.31	20.04	45.88
1350 °C	5.31	32.67	5.34	0.09	18.96	37.62
1450 °C	12.94	10.91	10.80	12.68	25.54	27.13
1550 °C	11.09	14.47	8.36	16.72	22.40	26.97

**Table 9:** Vickers hardness of (TiVNbTa)_2_AlC and relevant members.

	(TiVNbTa)_2_AlC (This Paper)	Ti_2_AlC [65]	V_2_AlC [66]	Nb_2_AlC [67]	Ta_2_AlC [68]
Vickers Hardness (GPa)	6.4	2.8	2.8	6.1	4.4

**Table 10:** Surface profile parameters of tracks at different temperatures (units: μm).

	R.T.	100 °C	200 °C	300 °C	400 °C
*R_a_*	0.31067	0.85650	0.23100	0.20600	0.47000
*R_q_*	0.39167	1.04450	0.28400	0.26350	0.59950
*R_z_*	1.64900	4.07400	1.08100	1.19150	2.33550
*R_v_*	0.80567	2.30800	0.54250	0.67750	1.15700
*R_z_*_1*max*_	2.31900	5.54400	1.92000	1.61050	3.39300

## Data Availability

The original contributions presented in this study are included in the article. Further inquiries regarding the data can be directed to the corresponding author.

## Nomenclature

*r*	atomic radius, Å
*x*	electronegativity
*VEC*	valence electron concentration
W	mass fraction, %
I	highest peak intensity, a.u.
$W_{R}$	wear rate, mm^3^/(N∙m)
w	wear scar width, mm wear thickness, mm
F	normal load, N
n	number of sample rotation cycles
*C*	elastic constants, GPa
*B*	bulk modulus, GPa
*E*	elastic modulus, GPa
*G*	shear modulus, GPa
*K_IC_*	fracture toughness, MPa × m^0.5^
*M*	brittle index, μm^−0.5^
*AU*	general anisotropy index AB compression anisotropy
*AG*	shear anisotropy
*Ra*	arithmetic mean deviation, μm
*Rq*	root-mean-square deviation, μm
*Rz*	maximum of height difference, μm
*Rv*	maximum of valley depth, μm
*Rz*1*max*	maximum of height difference within a single sampling length, μm
Greek symbols	
*ν*	Poisson’s ratio
Subscripts	
*i*	the certain phase in XRD patterns
